# Comparison of efficacy and safety of entecavir, tenofovir disoproxil fumarate, and tenofovir alafenamide fumarate treatment in patients with high viral load of hepatitis B virus

**DOI:** 10.1097/MD.0000000000043946

**Published:** 2025-08-29

**Authors:** Chenxia Zhang, Xinyue Chen, Zehua Hong, Yan Qi, Xudong Wang, Yaping Dai, Yuanwang Qiu

**Affiliations:** a Wuxi School of Medicine, Jiangnan University, Wuxi, Jiangsu, China; b Department of Infectious Diseases, Affiliated Wuxi Fifth Hospital of Jiangnan University, Wuxi, Jiangsu, China; c Department of Information Technology, Affiliated Wuxi Fifth Hospital of Jiangnan University, Wuxi, Jiangsu, China; d Department of Laboratory Medicine, Affiliated Wuxi Fifth Hospital of Jiangnan University, Wuxi, Jiangsu, China.

**Keywords:** antiviral therapy, chronic hepatitis B, high viral load, nucleos(t)ide analogue

## Abstract

Nucleos(t)ide analogues (NAs) have demonstrated potent efficacy in suppressing viral replication in chronic hepatitis B (CHB). This 48-week study compared the efficacy and safety of NA treatment for CHB patients with high viral load (hepatitis B virus [HBV] deoxyribonucleic acid [DNA] > 7 log_10_ IU/mL). This retrospective study included 180 nucleos(t)ide-naïve CHB patients with high viral load undergoing NA monotherapy, which were stratified into 3 groups: entecavir (ETV, n = 82), tenofovir disoproxil fumarate (TDF, n = 58), and tenofovir alafenamide fumarate (TAF, n = 40). The primary endpoint was the proportion of patients achieving HBV DNA < 20 IU/mL, with safety assessed by lipid profiles and renal function. The study subjects’ mean baseline HBV DNA levels were 7.68, 7.73, and 7.75 log_10_ IU/mL in ETV, TDF, and TAF groups, respectively. At week 48, TDF had a higher viral suppression (HBV DNA < 20 IU/mL) rate (67.74%) than TAF (37.50%) cohort (*P* = .004) and ETV (54.88%) was comparable to both (*P* > .05). Hepatitis B e antigen loss and seroconversion were comparable across all groups, with no hepatitis B surface antigen loss observed. Similar proportions of patients with high alanine aminotransferase levels at baseline achieved normalization by week 48 in all regimens. Low-density lipoprotein, total cholesterol, and triglyceride elevations were comparable across all groups. High-density lipoprotein reductions were 15.63%, 35.71%, and 17.86% in ETV, TDF, and TAF, respectively (*P* = .043), with TDF reducing high-density lipoprotein and total cholesterol more than ETV (*P* = .014 and *P* < .001). No significant differences were found in serum creatinine elevation or estimated glomerular filtration rates reduction among the groups. TDF was preferentially recommended for high viral load CHB patients, as its superior antiviral efficacy and comparable safety profile.

## 1. Introduction

Hepatitis B virus (HBV) infection persists as a significant global health concern, imposing substantial economic impact. Approximately 254 million individuals were chronically infected with HBV, with an annual increment of 1.2 million cases.^[1]^ Persistent HBV infection would lead to severe liver-associated complications, including decompensation, cirrhosis, and hepatocellular carcinoma (HCC).^[2–4]^ Higher baseline HBV deoxyribonucleic acid (DNA) levels are associated with higher risk of HBV recurrence, liver fibrosis, hepatocarcinogenesis, and liver-related mortality.^[5–8]^

As known, chronic hepatitis B (CHB) patients with potent and regular antiviral therapy through oral nucleos(t)ide analogues or pegylated interferon-alfa injection will achieve effective and durable viral replication suppression. This management effectively mitigates resistance development and liver-related complications.^[9,10]^ Nonetheless, the therapeutic efficacy and safety profiles of these agents in CHB patients with pretreatment high viral load (HVL) remain poorly characterized. At present, a unified definition of HVL in HBV infection remains elusive. However, the European Association for the Study of Liver 2017 Clinical Practice Guidelines on the management of the hepatitis B virus infection have delineated HVL as HBV DNA > 7 log_10_ IU/mL.^[11]^ Empirical evidence suggests that higher serum HBV DNA levels will lead to suboptimal virological response (VR), hepatitis B e antigen (HBeAg) seroconversion, and hepatitis B surface antigen (HBsAg) seroclearance in chronic HBV-infected individuals.^[11–13]^ Moreover, extensive research has confirmed that elevated baseline HBV DNA (> 4 log_10_ copies/mL) increases the incidence of liver fibrosis and HCC, and is responsible for the poorer prognosis and HCC recurrence after curative resection and orthotopic liver transplantation.^[6,14–17]^

Additionally, partially CHB patients with HVL receiving first-line drug therapy were characterized as low-level viremia (LLV) in clinical practice. A retrospective cohort containing 875 treatment-naïve CHB patients treated with entecavir (ETV) monotherapy, revealed that those with LLV at 5 years developed HCC more frequently than those who maintained VR^[18]^ (14.3% vs. 7.5%, *P *= .015). Consequently, treatment and management strategies for CHB patients with HVL present a complex and formidable challenge that requires increased clinical scrutiny.

ETV, tenofovir disoproxil fumarate (TDF), and tenofovir alafenamide fumarate (TAF) are recognized as first-line therapy choices recommended by the guidelines of EASL, the American Association for the Study of Liver Diseases (AASLD), and the Chinese Society of Hepatology. These agents serve as competitive inhibitors of HBV DNA polymerases, thereby suppressing viral replication.^[19–21]^ All aforementioned agents can achieve high rates of VR and alanine aminotransferase (ALT) normalization in HBeAg-positive and HBeAg-negative patients. Besides, they exhibit lower incidences of drug resistance compared to lamivudine in treatment-naïve patients and maintain favorable safety profiles over the long term.^[11]^ The purpose of this study was to evaluate the efficacy and safety of ETV, TDF, and TAF in CHB patients with HVL, defined as serum HBV DNA > 7 log_10_ IU/mL, to inform further clinical therapy management strategies, requiring heightened scrutiny and deliberation by the research and clinical communities.

## 2. Methods

### 2.1. Patients’ inclusion

**Figure 1.**
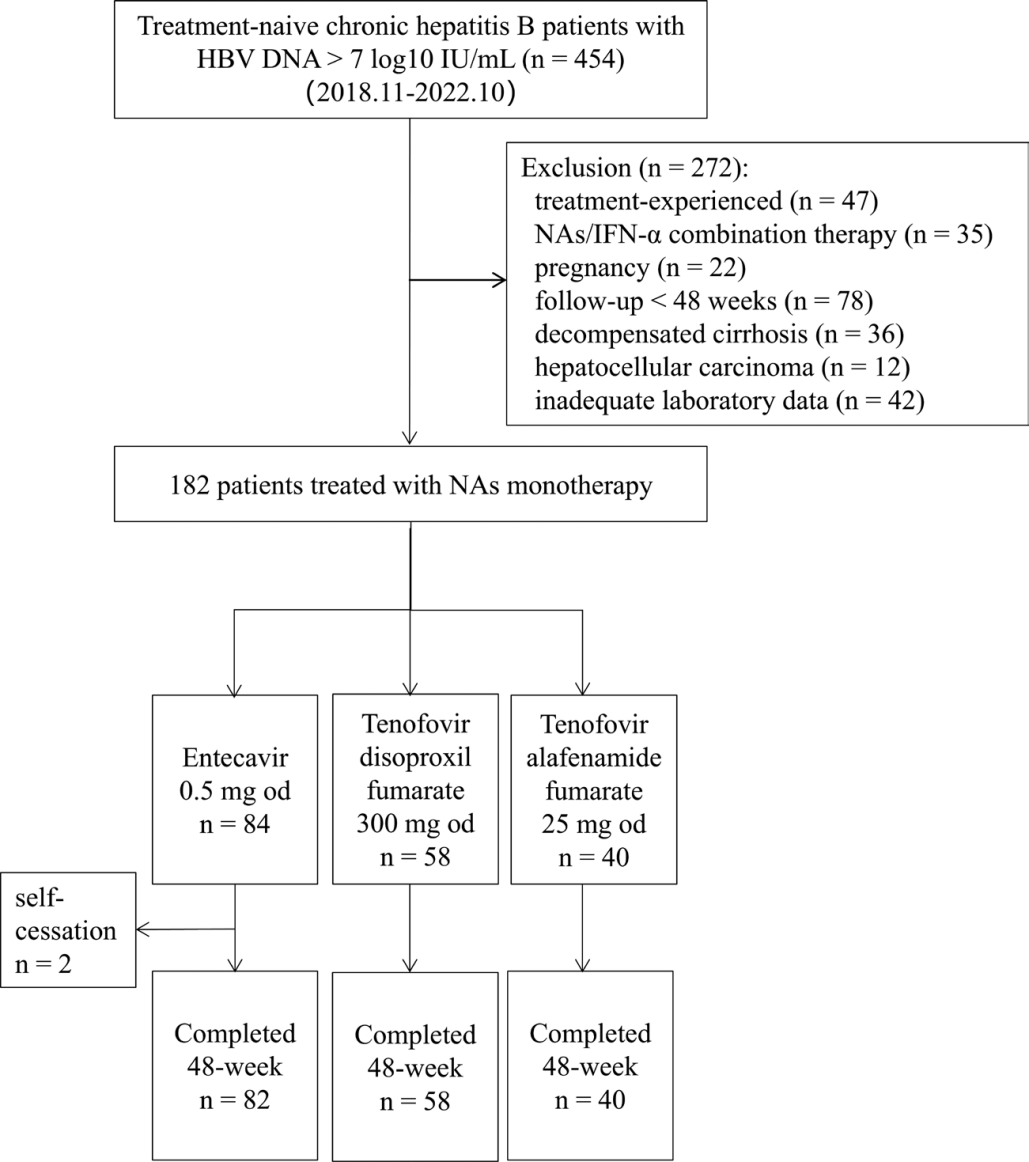
Patient flowchart. A total of 180 treatment-naive chronic hepatitis B patients treated with NAs monotherapy for 48 weeks were analyzed. HBV DNA = hepatitis B virus deoxyribonucleic acid; IFN-α = interferon α; NAs = nucleos(t)ide analogues; od = once daily.

The study included a total of 454 treatment-naive CHB patients with HVL who attended the Affiliated Wuxi Fifth Hospital of Jiangnan University (Wuxi, China) between November 2018 and October 2022. Patients’ screen eligibilities were as follows: (1) adults, aged 18 to 65 years, (2) chronic HBV infection, defined as HBsAg positive for at last 6 months, (3) HBV DNA > 7 log_10_ IU/mL at baseline, and (4) treatment-naïve and initiation nucleos(t)ide analogues monotherapy. Major exclusion criteria were the following: (1) co-infection with other hepatitis viruses or human immunodeficiency virus (HIV), (2) concomitant with autoimmune hepatitis, drug-induced hepatitis, or metabolic dysfunction-associated fatty liver diseases, (3) evidence of decompensated cirrhosis (such as ascites, variceal hemorrhage and so on) or HCC diagnosed via radiographic evaluation or liver biopsy, (4) comorbidities like hypertension, cardiopathy, diabetes mellitus, or nephropathy, (5) concurrent with other drugs (including immunomodulators, glucocorticoids, and cytotoxic drugs), (6) inadequate laboratory data, and (7) pregnancy or lactation. Finally, a total of 180 patients were enrolled for statistical analysis (Fig. 1). Of these, 6 patients in ETV and 3 patients in TAF with normal ALT received treatment based on: (a) significant necroinflammation (≥G2) on biopsy (n = 4), (b) family history of HBV-related cirrhosis (n = 4) or HCC (n = 1). This aligns with 2018 AASLD guidance recommending treatment for patients with high HBV DNA (>2000 IU/mL) plus evidence of significant liver disease.

### 2.2. Study design

This retrospective cohort study included patients treated with ETV, TDF, or TAF monotherapy for at least 48 weeks. Patients were divided into 3 groups according to antiviral agents: ETV group (n** **= 82, 0.5 mg once daily orally), TDF group (n** **= 58, 300 mg once daily orally), and TAF group (n** **= 40, 25 mg once daily orally), with the treatment endpoint measured at week 48. The primary efficacy endpoints were VR (HBV DNA < 20 IU/mL), ALT normalization, and HBeAg seroconversion. Safety outcomes were assessed by monitoring alterations in fasting lipid profiles and renal function.

### 2.3. Laboratory assessments

Laboratory assessments included hematological analysis (Sysmex, XN-10, Japan), serum biochemistry tests, and renal function measurement (Siemens, ADVIA 2400, USA). Real-time polymerase chain reaction assay (Roche Diagnostics, America ABI 7500 Real-Time PCR System, Hunan, China) was used to quantify serum HBV DNA. The situation of HBeAg, HBsAg, HBeAg antibodies (antibody to hepatitis B e antigen), and HBsAg antibodies (anti-HBs) were measured using a time-resolved immunofluorescence analyzer (Easycuta1260, Suzhou, China). Aspartate aminotransferase to platelet ratio index, fibrosis 4 score, and estimated glomerular filtration rate (eGFR)were calculated based on the following formulas:


$$\begin{array}{l} APRI\;=\frac{\frac{AST\;levels}{AST\;ULN\left(upper\;limit\;of\;normal\right)}}{Platelet\;count\left(10^{9}/L\right)}\times 100 \end{array}$$

$$\begin{array}{l} FIB-4=\frac{Age\left(years\right)\times AST\left(U/L\right)}{Platelet\;count\left(10^{9}/L\right)\times \sqrt{ALT\left(U/L\right)}} \end{array}$$

$$\begin{array}{l} eGFR=141\times \min{\left(Scr\;\left[mg/dL\right]/\kappa ,1\right)}^{\alpha }\;\\ \times max{\left(Scr/\kappa ,1\right)}^{-1.209}\times 0.993^{Age\;\left(years\right)}\;\\ \times 1.018\left[if\;female\right]\times 1.159\left[if\;black\right] \end{array}$$

The coefficient of κ was 0.7 and 0.9 for females and males respectively, while the value of α was −0.329 and −0.411 for females and males respectively. The terms min and max referred to the minimum and maximum of Scr/κ or 1.^[22]^ The upper limit of normal (ULN) for aspartate aminotransferase is 34 U/L.

### 2.4. Outcomes

The primary virological efficacy was evaluated based on VR, defined as serum HBV DNA levels < 20 IU/mL, and log_10_ reduction of HBV DNA levels. Additionally, LLV was defined as HBV DNA < 2000 IU/mL that was detectable after 48 weeks of antiviral treatment,^[23]^ and the percentage of LLV was the secondary virological efficacy. Biochemical response was defined as serum ALT levels ≤ ULN in the patients whose ALT levels > ULN at baseline according to the AASLD guidelines^[9]^ (ULN: 30 U/L for males and 19 U/L for females). Serologic response was a valuable endpoint in assessing the immune control situation of HBeAg-positive CHB patients before treatment, including the induction of HBeAg loss with or without antibody to hepatitis B e antigen seroconversion. In addition, another optimal indicator was the loss of HBsAg with or without anti-HBs existence. The safety assessment included changes in lipid parameters, the proportion of those presenting with dyslipidemia, changes in renal function, and the proportion of those with renal dysfunction.

### 2.5. Statistical analysis

Serum HBV DNA levels were log-transformed. First, distribution normality of continuous variables was determined using the Kolmogorov–Smirnov test and Shapiro–Wilk test. Descriptive statistics were used to characterize continuous variables, including all efficacy assessments. Continuous variables were described using mean [standard deviation (SD)] if normally distributed or median (interquartile range) if not normally distributed. Next, a 2-sided analysis of variance (ANOVA) or the Kruskal–Wallis test for independent samples was used for evaluation, and Bonferroni-adjusted *t* tests or Mann–Whitney *U* tests were performed for pairwise comparisons between groups (ETV vs TDF, TDF vs TAF, ETV vs TAF). Categorical variables were expressed as counts (%). Comparisons between treatment groups were conducted using a 2-sided Pearson Chi-Square or Fisher exact test. Statistical analyses were performed using SPSS 26.0 (IBM Corporation, Armonk, NY) and statistical significance was considered as *P* values <.05 (2-sided).

### 2.6. Ethical considerations

This study was performed in agreement with the principles of the Declaration of Helsinki and approved by the institutional review board of Affiliated Wuxi Fifth Hospital of Jiangnan University (No. 2024-028-1). Given the nature of retrospective analysis, the requirement for patients’ written informed consent was waived.

## 3. Results

### 3.1. Patients’ characteristics

A total of 182 CHB patients were included in the study, however, 2 patients in the ETV (self-cessation) did not complete the 48-week follow-up. Finally, a total of 180 CHB patients [82 (45.56%) receiving ETV treatment, 58 (32.22%) receiving TDF treatment, and 40 (22.22%) receiving TAF treatment] who matched the inclusion criteria were analyzed in this study. The demographics and baseline characteristics were similar between treatment arms (Table 1). The mean age of the patients was 35 years and approximately 62% of patients were male. The mean baseline HBV DNA levels were 7.68, 7.73, and 7.75 log_10_ IU/mL in the ETV, TDF, and TAF groups, respectively, whereas the difference was not statistically significant (*P *= .534). The median baseline ALT levels were 92.0, 76.5, and 78.0 U/L in the ETV, TDF, and TAF groups, respectively, and were comparable in all groups. In addition, the mean of serum creatinine (Cr), eGFR, and lipid parameters, and the median of aspartate aminotransferase to platelet ratio index and fibrosis 4 score at baseline were similar in the 3 groups.

**Table 1:** Patients’ demographics and baseline characteristics.

Characteristics	ETV (n* *= 82)	TDF (n* *= 58)	TAF (n* *= 40)	*P* value			
				ETV vs TDF vs TAF	ETV vs TDF	ETV vs TAF	TDF vs TAF
Age (years), mean (SD)	35.21 (8.83)	33.38 (7.37)	36.08 (8.82)	.251	.199	.611	.103
Gender, male, n (%)	51 (62.20)	34 (58.62)	26 (65.00)	.808	.670	.763	.524
HBV DNA (log_10_ IU/mL), mean (SD)	7.68 (0.35)	7.73 (0.38)	7.75 (0.40)	.534	.438	.291	.738
HBeAg status, n (%)				.993	.899	.980	.956
Positive	61 (74.39)	45 (77.58)	30 (75.00)				
Negative	7 (8.54)	4 (6.90)	3 (7.50)				
Unknown	14 (17.07)	9 (15.52)	7 (17.50)				
HDL (mmol/L), mean (SD)	1.25 (0.32)	1.24 (0.36)	1.25 (0.39)	.999	.967	.993	.969
LDL (mmol/L), mean (SD)	2.56 (0.76)	2.47 (0.77)	2.67 (0.69)	.446	.515	.440	.200
TC (mmol/L), mean (SD)	4.30 (0.76)	4.22 (0.74)	4.43 (0.74)	.391	.536	.368	.167
TG (mmol/L), mean (SD)	1.12 (0.43)	1.15 (0.57)	1.19 (0.54)	.768	.727	.438	.726
Albumin (g/L), mean (SD)	43.71 (3.22)	43.30 (4.01)	44.06 (4.55)	.614	.501	.666	.385
ALT (U/L), median (IQR)	92.00 (55.00, 217.25)	76.50 (49.50, 157.48)	78.00 (55.05, 319.75)	.777	.496	.965	.610
Normal ALT, AASLD guideline, *n* (%)	6 (7.32)	0 (0.00)	3 (7.50)	.068	.093	1.000	.065
AST (U/L), median (IQR)	65.00 (39.25, 124.00)	49.20 (31.50, 107.25)	51.00 (31.00, 144.25)	.441	.195	.606	.600
BUN (mmol/L), mean (SD)	4.69 (1.20)	4.39 (1.28)	4.76 (1.32)	.258	.152	.790	.170
eGFR (mL/min/1.73 m^2)^, mean (SD)	115.74 (10.91)	118.33 (10.75)	113.71 (12.27)	.124	.165	.358	.051
Serum creatinine (μmol/L), mean (SD)	64.92 (14.44)	63.19 (13.02)	66.95 (13.12)	.412	.470	.455	.165
PLT (×10^9^/L), mean (SD)	198.44 (54.05)	201.34 (42.75)	208.00 (46.33)	.600	.734	.339	.446
APRI, median (IQR)	0.90 (0.57, 1.94)	0.88 (0.48, 1.55)	0.77 (0.44, 2.20)	.529	.281	.495	.761
FIB-4, median (IQR)	1.15 (0.79, 1.78)	1.00 (0.68, 1.42)	1.09 (0.85, 1.61)	.224	.088	.560	.340

Variables are presented as mean (standard deviation) or median (interquartile range).

AASLD = America Association for the Study of Liver Diseases, ALT = alanine aminotransferase, APRI = aspartate aminotransferase to platelet ratio index, AST = aspartate aminotransferase, BUN *=* blood urea nitrogen, eGFR = estimated glomerular filtration rate; FIB-4, fibrosis 4 score, HBeAg *=* hepatitis B e antigen, HDL = high-density lipoprotein, IQR = interquartile range, LDL = low-density lipoprotein; n, number, PLT = platelet, SD = standard deviation, TC = total cholesterol, TG = triglyceride.

### 3.2. Efficacy

#### 3.2.1. Virological response

**Figure 2.**
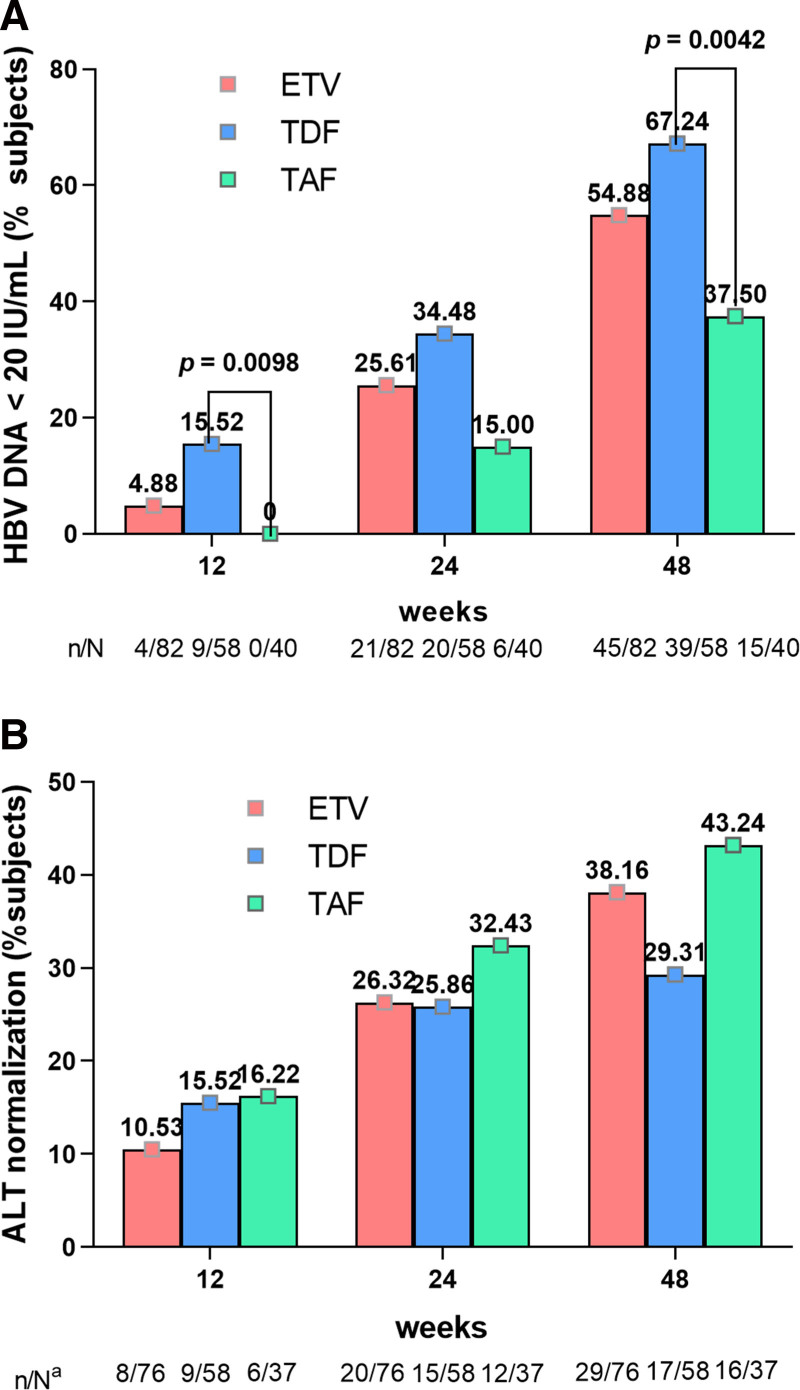
The proportions of patients achieved HBV DNA < 20 IU/mL (A) and ALT normalization (B) in ETV, TDF, and TAF groups at weeks 12, 24, and 48. ALT = alanine aminotransferase; ETV = entecavir; HBV DNA = hepatitis B virus deoxyribonucleic acid; TAF = tenofovir alafenamide; TDF = tenofovir disoproxil fumarate.

The proportions of patients achieving serum HBV DNA < 20 IU/mL at weeks 12, 24, and 48 were 4.88% (4/82), 25.61% (21/82), and 54.88% (45/82) in the ETV group, and 15.52% (9/58), 34.48% (20/58), and 67.24% (39/58) in the TDF group, and 0.00% (0/40), 15.00% (6/40), and 37.50% (15/40) in the TAF group (Fig. 2A). Statistically significant differences in VR rates at weeks 12 and 48 were observed between the 3 groups (*P* = .008 and *P *= .015, respectively). Further pairwise comparisons (Bonferroni-adjusted *t* tests) were then performed at week 48 to investigate the distinction between each other. The results demonstrated that the VR rates of the TDF group were higher than those of the TAF group (*P* = .004). In addition, those of the ETV group were similar to both the TDF and TAF groups (*P *= .141 and *P* = .071, respectively, and the adjusted significance level was 0.0167 using the Bonferroni method). All agents exhibited puissant antiviral efficacy with a considerable reduction in serum HBV DNA levels at 48-week (5.92 log_10_ IU/mL for ETV, 6.13 log_10_ IU/mL for TDF, and 5.78 log_10_ IU/mL for TAF; *P* = .080), and the details were described in Table 2. In addition, the proportion of patients in LLV (HBV DNA: 20–2000 IU/mL) status in the TAF group (n = 18, 45.00%) was higher compared to the ETV (n = 12, 14.63%) and TDF groups (n* *= 9, 15.52%) (*P* < .001).

**Table 2:** Summary of virological, serological, and biochemical response at week 48.

Virological, serological, and biochemical responses	ETV (n = 82)	TDF (n* *= 58)	TAF (n = 40)	*P* value			
				ETV vs TDF vs TAF	ETV vs TDF	ETV vs TAF	TDF vs TAF
HBV DNA < 20 IU/mL, n (%)	45 (54.88)	39 (67.24)	15 (37.50)	.015*	.141	.071	.004**
Reduction in HBV DNA, log_10_ IU/mL, median (IQR)	5.92 (3.64, 6.32)	6.13 (5.70, 6.43)	5.78 (5.11, 6.21)	.080	.114	.691	.064
HBV DNA status, n (%)				<.001***	.195	.001**	.004**
< 20 IU/mL	45 (54.88)	39 (67.24)	15 (37.50)				
20–2000 IU/mL	12 (14.63)	9 (15.52)	18 (45.00)				
>2000 IU/mL	25 (30.49)	10 (17.24)	7 (17.50)				
HBeAg loss, n/N† (%)	5/61 (8.20)	2/45 (4.44)	4/30 (13.33)	.384	.696	.691	.210
HBeAg seroconversion, n/N† (%)	4/61 (6.56)	2/45 (4.44)	2/30 (6.67)	.882	1.000	1.000	1.000
HBsAg loss, n/N‡ (%)	0/55 (0.00)	0/37 (0.00)	0/29 (0.00)	–	–	–	–
normal ALT, n/N§ (%) AASLD guideline	29/76 (38.16)	17/58 (29.31)	16/37 (43.24)	.348	.285	.604	.164

Variables are presented as median (quartile range).

AASLD = America Association for the Study of Liver Diseases, ALT = alanine aminotransferase, HBeAg = hepatitis B e antigen, HBsAg = hepatitis B surface antigen, n = number, N = number.

† Percentage of patients with HBeAg-positive at baseline.

‡ Percentage of patients with HBsAg positive at baseline.

§ Percentage of patients with elevated ALT at baseline to normal.

* *P *< .05.

** *P *< .01.

*** *P *< .001.

#### 3.2.2. Biochemical and serological response

Serum baseline ALT levels above ULN by AASLD guideline criteria were 92.68% (76/82) in the ETV group, 100.00% (58/58) in the TDF group, and 92.50% (37/40) in the TAF group. At week 48, the proportion of patients with ALT > ULN at baseline who achieved normalized ALT was 38.16% (29/76) in the ETV group, 29.31% (17/58) in the TDF group, and 43.24% (16/37) in the TAF group. The efficacy in ALT normalization was comparable across all 3 groups (*P* = .348) (Fig. 2B).

At week 48, 5 (8.20%) of the HBeAg-positive patients receiving ETV; 2 subjects receiving TAF (4.44%) and 4 (13.33%) patients in the TDF group achieved HBeAg loss; 8 patients (ETV: 4, TDF: 2, and TAF: 2) had HBeAg seroconversion. The differences between the 3 arms were not statistically significant (*P *= .384 and *P* = .882, respectively). In addition, both groups of patients who achieved HBeAg seroconversion had serum HBV DNA levels < 20 IU/mL at the same time (week 24). However, no one in either arm achieved HBsAg loss or seroconversion during the treatment period.

### 3.3. Safety

**Figure 3.**
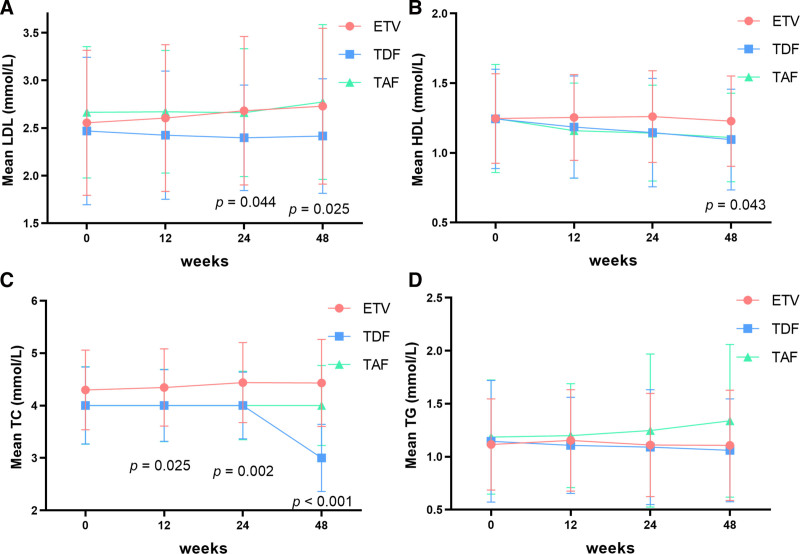
Changes in lipid profiles of patients treated with ETV, TDF, and TAF at weeks 12, 24, and 48. (A) LDL. (B) HDL. (C) TG. (D) TC. TDF may play an “lipid-lower” effect when compared to ETV and TAF. ETV: n = 82; TDF: n = 58; TAF: n = 40. LDL = low-density lipoprotein; HDL = high-density lipoprotein; TC = total cholesterol; TG = triglyceride; ETV = entecavir; TAF = tenofovir alafenamide; TDF = tenofovir disoproxil fumarate.

The majority of patients displayed good tolerance to all treatment regimens. At week 48, the proportions of patients with elevated low-density lipoprotein (LDL ≥ 3.4 mmol/L^[24]^) who had normal LDL at baseline receiving ETV were 10.29% (7/68). In contrast, the proportions among those who received TDF and TAF were 1.92% (1/52) and 10.81% (4/37), respectively. The proportions of patients with normal baseline high-density lipoprotein (HDL) in the ETV, TDF, and TAF groups presenting with HDL < 1.0 mmol/L at week 48 were 15.63% (10/64), 35.71% (15/42), and 17.86% (5/28), respectively. The proportions of patients with normal baseline triglyceride (TG) in the ETV, TDF, and TAF groups presenting with TG ≥ 1.7 mmol/L at week 48 were 8.22% (6/73), 9.80% (5/51), and 16.67% (6/36), respectively. The proportions of patients with normal baseline total cholesterol (TC) in the ETV, TDF, and TAF groups presenting with TC ≥ 5.2 mmol/L at week 48 were 8.70% (6/69), 0% (0/51), and 5.41% (2/37), respectively (Table 3). At week 48, the differences in the proportion of HDL reduction were significant (*P* = .043) and the results from Bonferroni-adjusted *t* tests suggested that the rates of abnormal HDL in TDF were higher than in ETV (*P* = .017, Bonferroni), but no differences were observed in the pairwise comparison between TAF and the others (Fig. 3). However, the percentages of elevated LDL, TG, and TC were similar (*P* = .162, *P* = .429, and *P* = .069, respectively) among the 3 regimens. Additionally, TDF was found to be associated with a decrease in HDL and TC levels in comparison with ETV (*P* = .009 and *P* < .001, respectively), as indicated by the lipid parameters’ fold change.

**Table 3:** Summary of laboratory abnormalities at week 48.

Changes	ETV (n = 82)	TDF (n* *= 58)	TAF (n* *= 40)	*P* value			
				ETV vs TDF vs TAF	ETV vs TDF	ETV vs TAF	TDF vs TAF
LDL, mmol/L							
Abnormal LDL, n/N† (%)	7/68 (10.29)	1/52 (1.92)	4/37 (10.81)	.162	.136	1.000	.156
Fold change, 48 wk/0 wk	1.10 (0.29)	1.02 (0.24)	1.05 (0.22)	.178	.078	.352	.453
HDL, mmol/L							
Abnormal HDL, n/N† (%)	10/64 (15.63)	15/42 (35.71)	5/28 (17.86)	.043*	.017*	.768	.105
Fold change, 48 wk/0 wk	1.00 (0.20)	0.90 (0.26)	0.91 (0.16)	.016*	.014*	.108	.836
TG, mmol/L							
Abnormal TG, n/N† (%)	6/73 (8.22)	5/51 (9.80)	6/36 (16.67)	.429	.759	.351	.536
Fold change, 48 wk/0 wk	0.96 (0.79, 1.25)	0.87 (0.69, 1.14)	1.09 (0.84, 1.47)	.073	.204	.071	.223
TC, mmol/L							
Abnormal TC, n/N† (%)	6/69 (8.70)	0/51 (0.00)	2/35 (5.41)	.069	.038*	.714	.163
Fold change, 48 wk/0 wk	1.04 (0.17)	0.94 (0.13)	1.02 (0.18)	.001**	<.001***	.420	.019*
Cr, μmol/L							

Changes	ETV (n = 82)	TDF (n* *= 58)	TAF (n* *= 40)	*P* value			
				ETV vs TDF vs TAF	ETV vs TDF	ETV vs TAF	TDF vs TAF
Abnormal Cr, n/N† (%)	0/82 (0.00)	0/58 (0.00)	0/40 (0.00)	–	–	–	–
Fold change, 48 wk/0 wk	1.02 (0.08)	1.02 (0.09)	1.03 (0.08)	.676	.740	.367	.585
eGFR, mL/min/1.73 m^2^							
eGFR < 90, n/N‡ (%)	0/81 (0.00)	0/56 (0.00)	0/38 (0.00)	–	–	–	–
Fold change, 48 wk/0 wk	0.99 (0.05)	0.98 (0.04)	0.98 (0.04)	.539	.518	.307	.611

Variables are presented as mean (standard deviation) or median (quartile range).

Fold change: the ratio of 48-week to baseline for the corresponding indicator.

Cr = serum creatinine, eGFR = estimated glomerular filtration rate, HDL = high-density lipoprotein, LDL = low-density lipoprotein, n = number, N = number, TC = total cholesterol, TG = triglyceride.

† Among patients with normal lipid parameters at baseline.

‡ Among patients with eGFR > 90 (mL/min/1.73 m^2^) at baseline.

* *P* < .05.

** *P* < .01.

*** *P *< .001.

**Figure 4.**
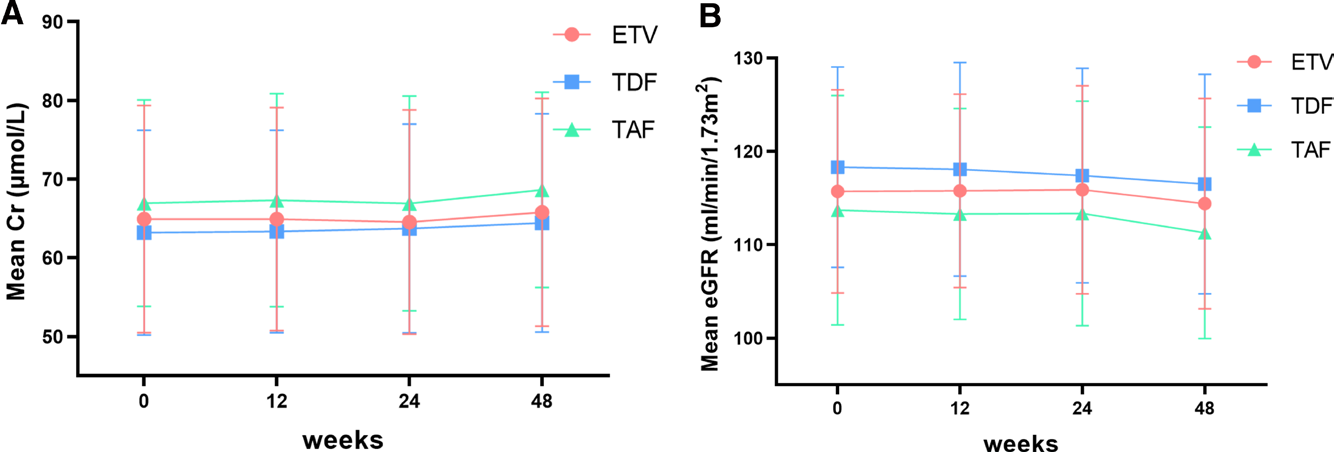
Changes in Cr (A) and eGFR (B) at week 12, 24 and 48. ETV: n = 82; TDF: n = 58; TAF: n = 40. Cr = serum creatinine; eGFR = estimated glomerular filtration rates; ETV = entecavir; TAF = tenofovir alafenamide; TDF = tenofovir disoproxil fumarate.

For renal function, no patient experienced an increase in Cr > 0.3 mg/dL (26.5 μmol/L) and no patient had an eGFR < 90 (mL/min/1.73 m^2^) among patients with normal renal function at baseline during treatment with the 3 regimens (Fig. 4). Additionally, no differences in Cr and eGFR were observed between the 3 agents.

## 4. Discussion

In this retrospective study, the efficacy and safety of oral antiviral agents (ETV, TDF, and TAF) were evaluated in CHB patients with HVL. At week 48, the inhibition of viral replication by ETV was comparable to that of TDF and TAF, but TDF demonstrated superior inhibition of viral replication in comparison to TAF. Moreover, the 3 agents presented considerably potent efficacy in reducing serum HBV DNA levels. Additionally, the occurrence of LLV was lower in the ETV and TDF groups than in the TAF group. The proportion of HBeAg and HBsAg loss and seroconversion was lower in all of the above agents. The comparable antiviral efficacy in HBV DNA suppression between ETV and TDF was consistent with the systemic review performed by Han et al.^[25]^ Furthermore, the difference between TDF and TAF in the proportions of HBV DNA < 20 IU/mL may be attributed to the higher percentage of patients in the immune tolerant phase in the TAF group.^[26]^ There was a minor percentage of patients achieving HBeAg loss and seroconversion and none exhibited serum HBsAg clearance, let alone anti-HBs production. Serological responses were similar in all above agents. It was difficult for current available antiviral regimens to achieve HBsAg loss and seroconversion, including ETV, TDF, and TAF.^[11]^ It is conceivable that the duration of the follow-up period was insufficient. This would suggest that the patient’s compromised immune system was not sufficiently robust to facilitate the elimination of pathogens. Furthermore, the heterogeneity of the study population may have a bearing on the efficacy of antiviral agents. Such factors as age, gender, and the differing natural histories of patients may be relevant in this regard.

As for the effect of ALT normalization, patients who had ALT > ULN at baseline achieved ALT normalization in the TDF group similar to the others. However, Agarwal et al identified that patients receiving TAF achieved ALT normalization significantly higher than those receiving TDF,^[21]^ according to the criteria recommended by the AASLD guideline at week 48. This variation might be explained by the heterogeneity of the populations studied and the different periods when the disease was present. It is acknowledged that ALT normalization could be influenced by a multitude of factors, including gender, body mass index, hepatic steatosis, and the presence of metabolic syndrome.^[27,28]^ Consequently, a longer observation period and more detailed demographic characteristics may be beneficial for monitoring ALT normalization and VR.

As known, ETV, TDF, and TAF were potent anti-HBV agents with high barriers to resistance and favorable safety profiles. It has been hypothesized that tenofovir (TFV) could impact lipid metabolism. At week 48, the proportion of patients receiving TAF who exhibited abnormal fasting lipid parameters was higher than those in the TDF and ETV groups. It is evident from the trend of alterations in fasting lipid parameters that TDF may potentially exert a lipid-lowering effect. In contrast, ETV and TAF appear to demonstrate a lipid-neutral response. Similarly, research focusing on weight changes in people living with HIV who switched from TDF to TAF showed that weight gain was approximately twofold during the initial 9 months after the switch to TAF compared to the previous state. This finding indicated that TAF exerts a distinct effect on weight gain.^[29]^ This weight gain may be associated with endocrine disorders, including diabetes mellitus and hyperlipidemia. In contrast, TDF treatment was regarded as having a lipid-lowering effect in patients infected with HIV.^[30]^ Interestingly, it was still unknown how TDF reduced HDL, TG, and TC in the meantime. Furthermore, a recent study showed that peripheral blood mononuclear cells treated with TAF tended to utilize glucose or pyruvate rather than fatty acid to power their mitochondria compared to those treated with TDF, and TAF was associated with the reduction of mitochondrial adenosine triphosphate production.^[31]^ Accordingly, the other energy supply materials, including fatty acids, were accumulated in the cells. This might illustrate the different roles of TAF and TDF in lipid metabolism in CHB patients.

Renal function was assessed by eGFR and Cr at week 48, including the proportion of eGFR < 90 mL/min/1.73 m^2^ and changes in eGFR and Cr. The results indicated that ETV, TDF, and TAF could impact patients’ renal function, but no difference was observed between the 3 agents. This finding was consistent with the observations reported in previous studies and clinical practice.^[32]^ Both TDF and TAF are prodrugs of TFV, the mechanism of the decrease in renal function may be due to the accumulation of TFV in the proximal tubular cells, which subsequently results in direct injury to the proximal tubular cells and mitochondria, ultimately leading to proximal tubular dysfunction and tubular cells necrosis.^[33,34]^ Previous studies had demonstrated that TAF was less nephrotoxic than TDF, the slight difference between our study was mainly due to the limited follow-up period and the small sample size. Consequently, longer follow-up and larger sample studies are needed to understand the long-term effect on renal function changes after TDF and TAF treatment.

There are several limitations in this study. First, patients’ data was less sufficient than in prospective studies, and some demographics and baseline characteristics were indeterminate, leading to selection bias. Moreover, the smaller scale and the shorter duration of follow-up could not evaluate the long-term effects of antiviral agents on CHB patients with HVL. Increasing the sample size, prolonging follow-up duration, and carrying out propensity score matching to control confounding factors may make a difference.

## 5. Conclusion

In conclusion, our study revealed that ETV, TDF, and TAF had potent antiviral efficacy in CHB patients with HVL over 48 weeks of treatment. The anti-HBV potency of ETV was comparable to that of TDF and TAF, but that of TDF was superior to TAF. As with previous studies, the rates of HBeAg and HBsAg loss and seroconversion remained low. Long-term treatment is required to observe serological response. The proportions of ALT normalization were similar in ETV, TDF, and TAF arms. The safety profiles of TDF were comparable to TAF and ETV, with fasting lipid abnormalities and renal function changes still being under control. Consequently, TDF was preferentially recommended for HVL CHB patients. Of course, longer duration of follow-up and larger dimension studies are required to investigate the antiviral efficacy and safety of oral antiviral agents in CHB patients with HVL.

## Acknowledgments

The authors would like to thank all participating volunteers for their contributions to this work.

## Author contributions

**Conceptualization:** Yuanwang Qiu.

**Formal analysis:** Xinyue Chen.

**Investigation:** Zehua Hong, Yan Qi.

**Methodology:** Xudong Wang, Yaping Dai.

**Writing – original draft:** Chenxia Zhang.

**Writing – review & editing:** Chenxia Zhang.
